# Study of the Dispersion Compensation Double-Layer Diffractive Optical Components Based on Metasurface and Grating, and Their Application in Augmented Reality Displays

**DOI:** 10.3390/ma17215291

**Published:** 2024-10-30

**Authors:** Jiahang Zhang, Siqi Liu, Wei Zhang, Sijia Jiang, Ding Ma, Liang Xu, Mingyu Yang, Qingbin Jiao, Xin Tan

**Affiliations:** 1 Changchun Institute of Optics, Fine Mechanics and Physics, Chinese Academy of Sciences, Changchun 130033, China; zhangjiahang17@mails.ucas.ac.cn (J.Z.);; 2 University of Chinese Academy of Sciences, Beijing 100049, China; 3 Center of Materials Science and Optoelectronics Engineering, Chinese Academy of Sciences, Beijing 100049, China

**Keywords:** metasurface, light shaping, dispersion compensation, double-layer diffractive optical element, diffractive waveguide, augmented reality

## Abstract

We employed a double-layer coupled diffractive optical element, based on metasurfaces and diffraction gratings, which exhibits wavefront modulation and chromatic dispersion compensation. Utilizing this double-layer coupled diffractive optical element in the optical information transmission process of a diffractive waveguide allows for the transmission of color image information using a single-layer waveguide structure. Our results demonstrate that, under the conditions of a field of view of 47° × 47°, an entrance pupil size of 2.9 × 2.9 mm^2^, and an exit pupil extension size of 8.9 mm, the uniformity of the brightness for each monochromatic field reached 85%, while the uniformity of color transmission efficiency exceeded 95%.

## 1. Introduction

Metasurface structural units can impart a phase shift of ∆ϕ to the incident light. This phase shift relates to the wavelength λ of the incident light and the structural parameters of the metasurface units, including the substrate material, unit material, shape, and dimensions such as geometric phase elements’ phase modulation. One can establish different parameters for structural units at each position to achieve specific phase responses. Currently, many metasurface designs utilize this method for optical information transmission and imaging optics [1,2,3,4,5,6,7,8,9,10,11,12,13,14,15,16,17,18,19,20,21,22,23,24,25,26,27,28,29,30]. It can achieve broadband achromatic focusing and imaging in the visible range, with design efficiency reaching 90% for large NAs such as 0.8 [31,32,33,34,35].

Augmented reality displays primarily integrate real-world scenes with virtual images from miniature displays through diffractive optic waveguide combiners. These diffractive waveguides exhibit excellent characteristics, such as a wide field of view, lightweight design, and extensive eye-movement range. To achieve enhanced augmented reality effects with a larger field of view and improved color representation, numerous scholars have conducted extensive and in-depth research. However, current limitations persist regarding the field of view and significant deviations in imaging quality at wide angles. This issue arises because the existing multi-color diffractive waveguides typically utilize multi-layer waveguide structures to transmit the light information of different colors separately. For instance, red, green, and blue light may each be transmitted through three distinct layers, or red and green light can use two layers for their respective transmissions [36,37,38,39]. Currently, diffractive waveguides often deploy surface-relief gratings, volume holographic gratings, and polarization holographic volume gratings as coupling elements for in-coupling and out-coupling [30,40,41,42,43,44,45]. However, when faced with multi-field angle (wide angular spectrum) and multi-color light (multispectral) coupling information, a single diffractive grating cannot adequately meet the simultaneous in-coupling and out-coupling demands of multi-spectral and multi-angular requirements. In this context, metasurfaces, due to their flexible phase responses, can offer a more complex modulation of optical field information compared to traditional diffractive gratings [30,46,47,48,49,50,51,52,53]. This capability positions metasurfaces as the in-coupling and out-coupling elements of diffractive waveguides.

In this study, we developed a double-layer coupled diffractive optical element (DOE) by integrating one-layer metasurface and one-layer diffraction grating structures. We leveraged the opposing dispersion phenomena generated by these components to achieve chromatic aberration correction. This innovation enhances light information coupling in augmented reality diffractive waveguides. Furthermore, the use of a monolayer waveguide structure facilitated the transmission of light information. By designing the wavefront modulation capabilities of the metasurface, we stabilized the beam size during the light information transmission process within the diffractive waveguide. This effectively transformed the originally complex beam size variations into fixed-size beams for the effective propagation of light information. During the in-coupling and out-coupling process, we employed a similar bilayer structure for efficient light information exit and wavefront reconstruction. Consequently, we achieved a light information transmission efficiency of 7.5% at 450, 532, and 632 nm, with a brightness uniformity of 99.3–99.9%. The uniformity of the total exit pupil extension reached 97%. The uniformity of the efficiency reached 85% within a diagonal field of view of 74 degrees.

## 2. Materials and Methods

### 2.1. Phase Response of Meta-Atom Structures

**Figure 1:**
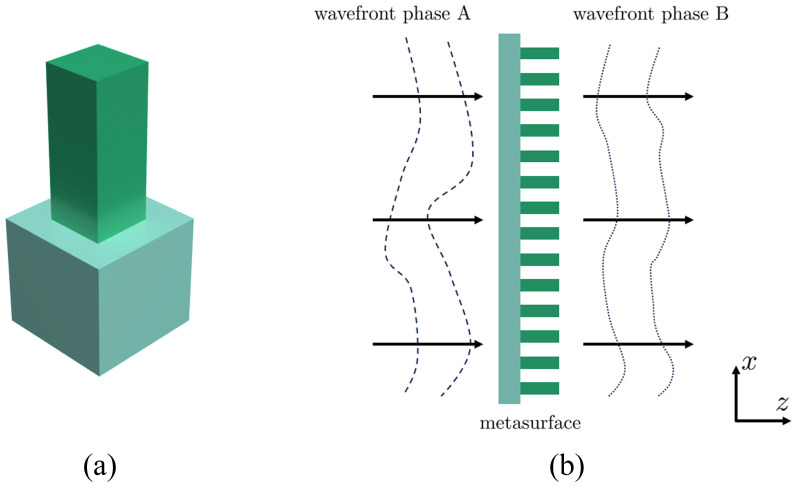
Diagram of the metasurface of double-layer coupled DOE: (**a**) metasurface cell structure; (**b**) light-shaping process of metasurface of the wavepropagation along z direction.

Figure 1 illustrates the meta-atom structure of the metasurface, including a substrate and a square pillar. This could achieve different phases of modulation with different pillar heights and widths. This achieves the desired modulation of the optical field phase by periodically arranging square pillar structures of varying sizes on the substrate material. By altering the height and side length of the pillar structures, one can adjust the phase modulation values at different positions. In the application design, the overall propagation direction of the incident light is along the z-direction, as shown in Figure 1.

**Figure 2:**
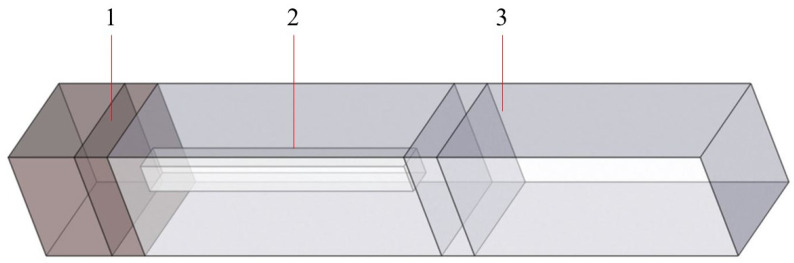
Structure of metasurface cells for simulation in COMSOL with 1 representing the substate, 2 representing the pillar, and 3 representing the air cavity.

**Figure 3:**
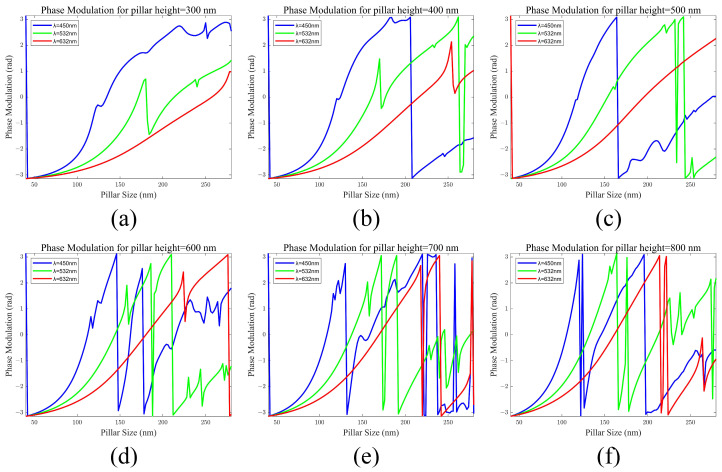
Phase modulation of a metasurface cell with different pillar heights. (**a**) h = 300 nm (**b**) h = 400 nm (**c**) h = 500 nm (**d**) h = 600 nm (**e**) h = 700 nm (**f**) h = 800 nm.

By varying the width (a) and height (h) of the cylindrical structures, we can achieve different phase responses in localized positions. We utilized COMSOL 6.1 software to perform simulation calculations and analyses of the metasurface unit structures employing the Finite Element Method (FEM). The periodic structure of the metasurface was set at 320 nm, with the initial cylinder height set at 400 nm. The actual simulation structure of the metasurface unit is depicted in Figure 2. The input field is set as the plane wave at the left plane. The substrate material is SiO_2_ and the pillar material is TiO_2_. To consider the phase generated by the pillars within the metasurface unit and to ensure stability after transmitting over a shorter distance within the air cavity, we set the analysis height of the unit to 800 nm. The amplitude and phase response of the field at the right plane was used for the design. The pillar material was TiO_2_, of which the refractive index was 2.5437, 2.4367, and 2.3749 at wavelengths of 450 nm, 532 nm, and 632 nm, respectively. The substrate material was set as SiO_2_ with the refractive index set as 1.5. This adjustment prevents phase coupling instability from affecting the accuracy of the simulation results. By adjusting the cylinder width, we analyzed the phase control functions for incident light wavelengths of 450 nm, 532 nm, and 632 nm, as shown in Figure 3. The results indicate that adjusting the cylinder width allows for different phase control functions for incident light of varying colors. According to the multi-wavelength metasurface design, the meta-atoms must achieve phase modulation in the range of [0, 2π]. The phase modulation for wavelengths of 632 nm cannot satisfy the conditions at pillar heights h = 300 nm, 400 nm, 500 nm, and 600 nm. Under the condition of h ≥ 700 nm, it is possible to achieve phase modulation within the range of [0, 2π] for light wavelengths of 450 nm, 532 nm, and 632 nm simultaneously.

### 2.2. Metasurface Design Theory

Metasurfaces could achieve different phase modulation for several wavelengths at the same time. For example, in the metasurface design for three wavelengths $\lambda =\left[\lambda_{1},\lambda_{2},\lambda_{3}\right]$, the target phase modulation could be written as $\Delta \psi \left(\rho ,\lambda_{j}\right)$, with j=1,2,3, $\rho =\left(x,y\right)$. In the case where only the phase modulation is considered, without consideration of the amplitude modulation, the target filed modulation could be expressed as follows:

(1) $$T_{\mathrm{ideal}}\left(\rho ,\lambda_{j}\right)=e^{i\cdot \Delta \psi \left(\rho ,\lambda_{j}\right)}$$

where “i” represents the imaginary unit. In multi-wavelength phase modulation, the efficiency of each wavelength could be changed by varying the design weight $W_{j}$ of each wavelength. The constant phase deviation $\Delta \psi (\lambda_{j})$ need to be calculated to satisfy the phase modulation for multiple wavelengths at the same time. Using the optimization due to the maximum project value, the $\Delta \psi (\lambda_{j})$ is calculated. Then, the phase modulation could be expressed as

(2) $$T_{\mathrm{ideal}}\left(\rho ,\lambda_{j}\right)=e^{i\cdot [\Delta \psi (\rho ,\lambda_{j})+\Delta \psi (\lambda_{j})]}$$

**Figure 4:**
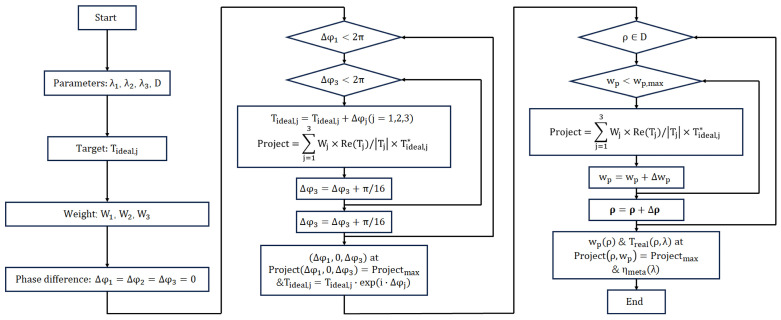
Workflow of multi-wavelength phase modulation metasurface design.

The design workflow for the three-wavelength design is shown in Figure 4.

### 2.3. The Dispersion Compensation Principle of Dual-Layer Coupled Diffractive Optical Elements

Different wavelengths of light, at the same incident angle, exhibit varying diffraction angles. If we modify the incident angles of different wavelengths entering a diffraction grating using a metasurface modulating device, we can ensure that the diffraction angles for multiple colors of light remain identical. This adjustment helps to eliminate angular deviations during the coupling multi-color information, thereby addressing the phenomenon of dispersion in optical waveguide information transmission.

**Figure 5:**
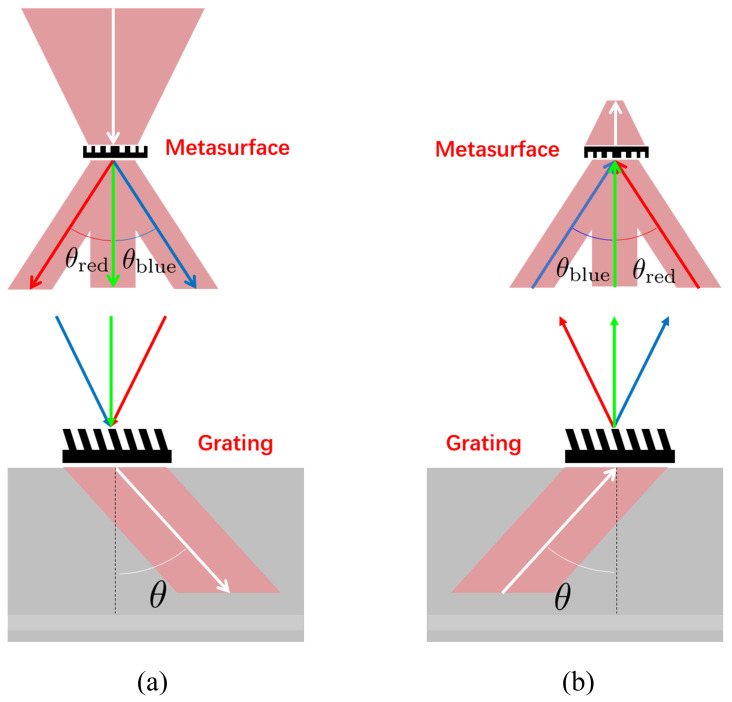
Dispersion compensation double-layer diffractive optical component based on metasurface and grating (red, blue, green arrows represents the propagation direction of red, blue, green beams respectively, white arrows represents the propagation direction of red, blue, green beams are same): (**a**) forward light shaping process; (**b**) backward light shaping process [54].

This method combines metasurfaces and diffraction gratings to form a dual-layer diffraction optical element. Each layer has its own dispersion effect due to diffraction. When the dispersion effects from the metasurface layer oppose those from the diffraction grating layer, the overall dispersion impact of the dual-layer optical element is neutralized. Through the dispersion compensation of these dual layers, we achieve the elimination of dispersion phenomena during the coupling of optical information into and out of the optical waveguide, as illustrated in Figure 5.

For example, consider the case of red, green, and blue light beams that are incident perpendicularly on a grating. The grating equation is as follows:

(3) $$n\left(\lambda \right)\cdot d\cdot \sin\left(\theta_{+1}\right)=+1\cdot \;\lambda$$

where n(λ) is the refractive index at wavelength λ, while the diffractive angle of +1 order could be written as follows:

(4) $$\theta_{+1}\left(\lambda \right)=\mathrm{asin}\left(\frac{\lambda }{n\left(\lambda \right)\cdot d}\right)$$

Equation (4) shows that the diffractive angle of +1 order for red, green, and blue light cannot be same as the same incident angle. However, it was shown that the diffractive angle of the three wavelengths is the same ($\theta_{R,+1}=\theta_{G,+1}=\theta_{B,+1}$) when using red, green, and blue light with different incident angles $\theta_{\mathrm{in}}\left(\lambda \right)$. The angles should satisfy the grating equation:

(5) $$n\left(\lambda \right)\cdot d\cdot \sin\left(\theta_{+1}\right)-n_{\mathrm{air}}\cdot d\cdot \sin\left(\theta_{\mathrm{in}}\left(\lambda \right)\right)=+1\cdot \lambda$$

where $\theta_{+1}$ is the common diffractive angle for three wavelengths. The incident angle of different wavelengths $\theta_{\mathrm{in}}\left(\lambda \right)$ could be written as follows:

(6) $$\theta_{\mathrm{in}}\left(\lambda \right)=\mathrm{asin}\left(\frac{n\left(\lambda \right)\cdot d\cdot \sin\left(\theta_{+1}\right)-\lambda }{n_{\mathrm{air}}\cdot d}\right)$$

When the meta-surface couples with multi-colored light, it modulates the propagation angle of the red light to $\theta_{\mathrm{in}}\left(\lambda_{R}\right)=\mathrm{asin}\left(\frac{n\left(\lambda_{R}\right)\cdot d\cdot \sin\left(\theta_{+1}\right)-\lambda_{R}}{n_{\mathrm{air}}\cdot d}\right)$, the propagation angle of green light to $\theta_{\mathrm{in}}\left(\lambda_{G}\right)=0^{o}$, and the propagation angle of blue light to $\theta_{\mathrm{in}}\left(\lambda_{B}\right)=\mathrm{asin}\left(\frac{n\left(\lambda_{B}\right)\cdot d\cdot \sin\left(\theta_{+1}\right)-\lambda_{B}}{n_{\mathrm{air}}\cdot d}\right)$. Under this wavefront modulation effect, and by integrating the light modulation effects of the meta-surface layer and the grating layer, the three colors of light are modulated to propagate at the same angle within the medium.

### 2.4. Application of Chromatic Aberration-Corrected Dual-Layer Coupled Diffractive Elements in Diffractive Waveguide Combiners

**Figure 6:**
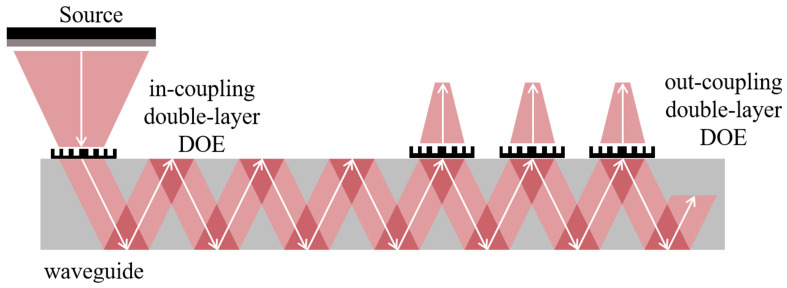
Light propagation process in the x-domain in a metasurface-based double-layer diffractive waveguide with white arrow shows the propagation direction of beams [54].

**Figure 7:**
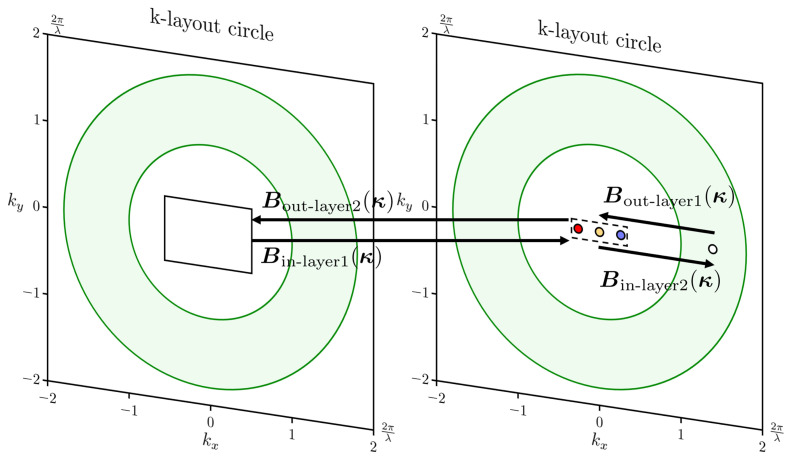
Light propagation process in the k-domain in a metasurface-based double-layer diffractive waveguide [54].

In geometric space, the process of diffraction optical waveguide information transmission and coupling using an achromatic double-layer coupled diffraction optical element is illustrated in Figure 6. By employing this achromatic double-layer coupled diffraction optical element as a coupling component for the optical waveguide combinator, the optical information transmission process within the wave vector space is depicted in Figure 7. The wavefront phase decides the propagation of optical information. The wavefront phase is modulated after in-coupling and reconstructed after out-coupling in the diffractive waveguide. In this modeling, the light information is replicated to the spatial position of the out-coupling area and continues to propagate. This process ensures that the light information generated by the microscale light source propagates into the human eye after out-coupling through the diffractive waveguide to enable an augmented reality display.

From the perspective of optical coupling, the achromatic double-layer coupled structure effectively reduces the angular spectral range that requires optimization for the coupling grating. This, in turn, decreases the area of optical information in the vector space, thereby lowering the design requirements for the coupling grating. Furthermore, it enables the transmission of light information across three colors within the same layer of the optical waveguide, significantly reducing the volume of the diffractive optical waveguide and promoting lightweight design.

There is another requirement for the application of double-layer DOEs in diffractive waveguides. In digital displays, a color image can be viewed as being composed of three separate components of light: red, green, and blue. These three components are transmitted independently and do not interfere with one another. Therefore, to ensure that the output light information retains the same color composition as the incident light information before coupling, it is necessary to achieve equal coupling efficiencies for all three-color components. This requirement is manifested in the analysis process as the high uniformity of the in-coupling and out-coupling for the three-color components of light.

## 3. Design, Results, and Analysis

### 3.1. Design of Double-Layer Diffractive Optical Element Structures

**Figure 8:**
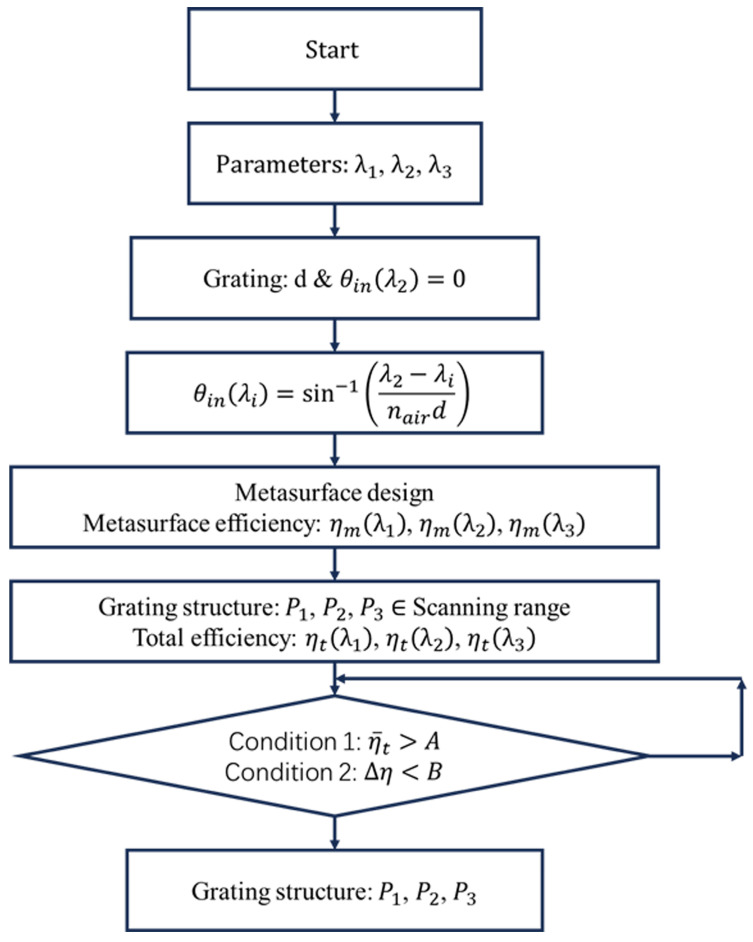
Workflow of metasurface-based double-layer DOE design.

Based on the functional design of dispersion compensation double-layer coupled diffraction waveguides, the specific design flowchart is illustrated in Figure 8. The metasurface design process is introduced in Section 2.2. First, determine the beam transmission angle $\theta_{\mathrm{wg}}$ within the waveguide. Then, backward-calculate the grating period d of the second-layer coupling grating structure and the incident angles for three color wavelengths $\theta_{\mathrm{in}}\left(\lambda_{R}\right)$ $\theta_{\mathrm{in}}\left(\lambda_{G}\right)$ $\theta_{\mathrm{in}}\left(\lambda_{G}\right)$. Subsequently, establish the phase control for the first-layer coupling metasurface corresponding to the three wavelengths, as follows:

(7) $$\begin{array}{c} ∆\psi \left(\lambda_{R}\right)=2\pi x\cdot \sin\left(\theta_{\mathrm{in}}\left(\lambda_{R}\right)/\lambda_{R}-\psi_{\mathrm{in}}\left(\lambda_{R}\right)\right)\\ ∆\psi \left(\lambda_{G}\right)=2\pi x\cdot \sin\left(\theta_{\mathrm{in}}\left(\lambda_{G}\right)\lambda_{G}-\psi_{\mathrm{in}}\left(\lambda_{G}\right)\right)\\ ∆\psi \left(\lambda_{B}\right)=2\pi x\cdot \sin\left(\theta_{\mathrm{in}}\left(\lambda_{B}\right)\lambda_{B}-\psi_{\mathrm{in}}\left(\lambda_{B}\right)\right) \end{array}$$

Through the design of specific metasurface structures, we sought to optimize the coupling efficiency in dichroic wave interactions. This culminated in the design of a bilayer coupling diffractive optical element. We defined the incident phase as a spherical phase, with wavelengths set at 450 nm, 532 nm, and 632 nm. The refractive index of the waveguide was given as 1.80, with a grating period of d = [450 nm, 500 nm] for coupling incident and outgoing light. We selected the height of the nanopillars in the metasurface unit as 800 nm. Based on the mapping relationship between the phase response of the metasurface unit structures and the width of the pillars, we proceeded with the arrangement design of the metasurface unit structures to achieve the aforementioned phase modulation. The required angles of incident light for modulation at different grating periods are detailed in Table 1.

By analyzing the coupling efficiency of three-color light under the varying design weights of three wavelengths and varying grating periods, we selected the weights as W_1_ = 1.70, W_2_ = 0.98, and W_3_ = 1.90 for the metasurface design and structure, with a grating period of 500 nm, an inclination angle of 25°, and a grating depth of 580 nm as the coupling input diffraction grating structure. With this grating structure, the efficiencies of the in-coupling metasurface are 69.26%, 49.96%, and 70.92% at wavelengths of 450 nm, 532 nm, and 632 nm, respectively, and the efficiencies of the in-coupling grating are 68.38%, 94.63%, and 66.36% at wavelengths of 450 nm, 532 nm and 632 nm, respectively. Thus, the total in-coupling efficiencies are 47.36%, 47.28%, and 47.06% for wavelengths of 450 nm, 532 nm, and 632 nm, respectively. The in-coupling uniformity of the three wavelengths is 99.68% when using the equation of uniformity [55]:

(8) $$U=1\;-\;\frac{I_{\max}-\;I_{\min}}{I_{\max}+I_{\min}}$$

**Figure 9:**
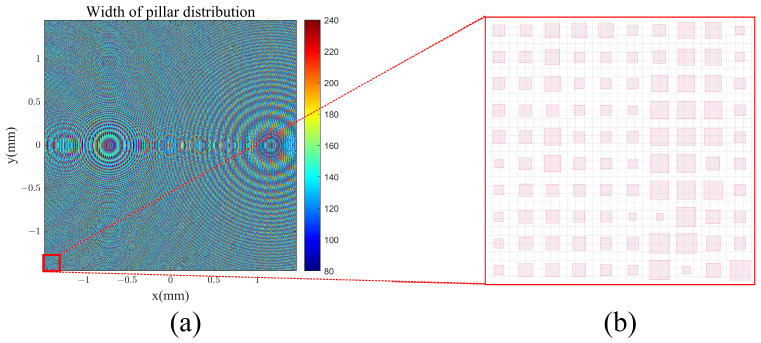
In-coupling metasurface layer: (**a**) width of pillar size distribution with units given as nm; (**b**) local structure of metasurface at bottom left corner.

**Figure 10:**
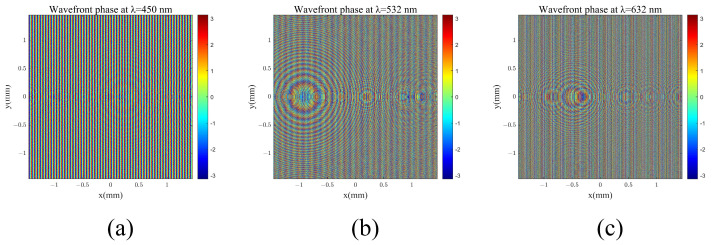
Phase of field coupled by in-coupling double-layer diffractive optical elements: (**a**) λ = 450 nm; (**b**) λ = 532 nm; (**c**) λ = 632 nm.

Figure 9 illustrates the arrangement of structural units in the coupling input metasurface. Different colors represent varying unit structure dimensions. The phase control results for the coupling of incident light at three distinct wavelengths are displayed in Figure 10.

**Figure 11:**
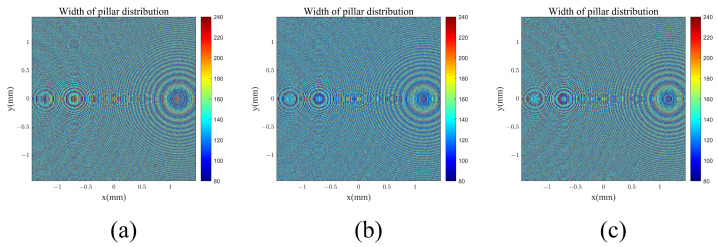
Out-coupling metasurface width of pillar size distribution with unit as nm. (**a**) first out-coupling (**b**) second out-coupling (**c**) third out-coupling.

**Figure 12:**
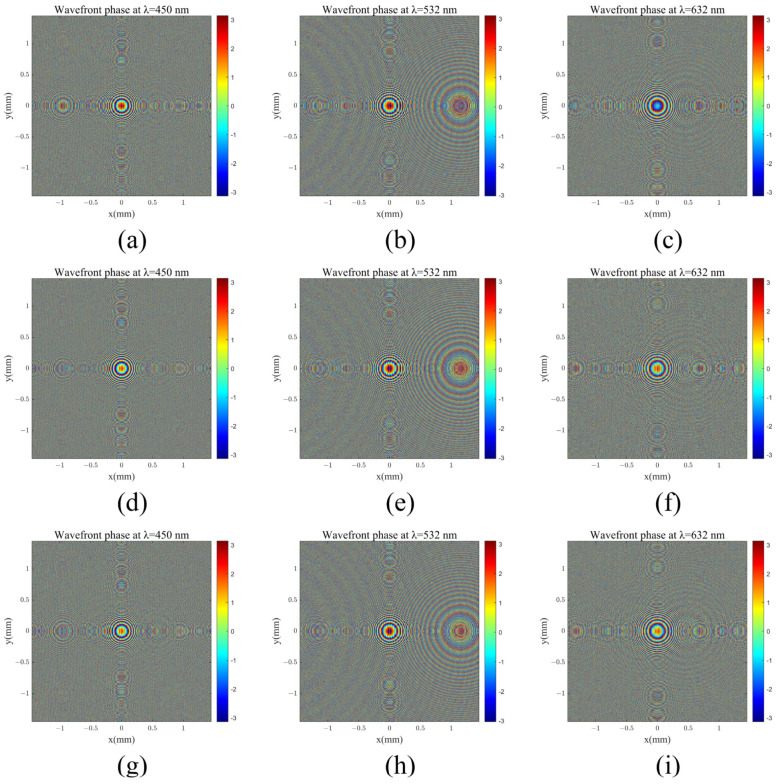
Phase of field coupled by three out-coupling double-layer diffractive optical elements: (**a**–**c**) λ = 450 nm; (**d**–**f**) λ = 532 nm; (**g**–**i**) λ = 632 nm.

Similarly, by evaluating the coupling efficiency of three-color light with different grating periods, we chose a configuration with a grating period of 500 nm, fill factor of 69% and grating depth of 490 nm, fill factor of 66%, and grating period of 470 nm, fill factor of 36% and grating depth 44 of 320 nm as the three out-coupling diffraction grating structures, respectively. Figure 11 presents the arrangement of structural units in the coupling input metasurface. The pillar distribution is calculated using the design workflow at three wavelengths design shown in Figure 4. The weights of the design for three wavelengths were W_1_ = 1.10, W_2_ = 1.03 and W_3_ = 1.20, W_1_ = 2.00, W_2_ = 1.19 and W_3_ = 1.15, W_1_ = 1.21, W_2_ = 1.10, and W_3_ = 1.17 for the first, second, and third out-couplings, respectively. The phase control results for the coupling of incident light at these three wavelengths are shown in Figure 12. This was simulated using the point-to-point complex field response of meta-atom shown in Figure 11, with the incident beam as a plane wave with a special angle ($\theta_{\mathrm{in}}$ (450 nm) = 9.44°, $\theta_{\mathrm{in}}$ (532 nm) = 0°, $\theta_{\mathrm{in}}$ (632 nm) = −11.54°). The three angles are the design angles after coupling with the grating layer of the out-coupling double-layer DOEs. All the uniformity results were calculated using Equation (6). The structures of all the double-layer DOEs are shown in Table 2. All the efficiencies of the out-coupling gratings and out-coupling metasurfaces are shown in Table 3. The uniformity of the first, second, and third out-coupling DOEs for the three wavelengths are 99.92%, 99.44%, and 99.72%, respectively. For all three out-coupling DOEs, the uniformity of the total out-coupling efficiency is 97.27%.

### 3.2. Phase Modulation Analysis of Double-Layer Diffractive Optical Elements

Therefore, the propagation of optical field $E_{⊥}(x,z_{0})$ with the propagation distance ∆z can be clearly shown by the following calculation:

1. Using Fourier transform for field at 0 plane $E_{⊥}(x,z_{0})$ the information in spatial-frequency domain ${\tilde{E}}_{⊥}(k_{x},z_{0})=F[E_{⊥}(x,z_{0})]$ can be obtained.
2. Multiply the result with the propagation function with propagation distance Δz in the spatial-frequency domain $exp(i\cdot k_{z}\Delta z)$, where $k_{z}=\sqrt{k^{2}-k_{x}^{2}}$. The information in the spatial-frequency domain at target plane is $z=z_{0}+∆z$ is ${\tilde{E}}_{⊥}(k_{x},z)exp(i\cdot k_{z}\Delta z)$.
3. Use inverse Fourier transform for the field at target plane $z=z_{0}+∆z$ to obtain the field information in the space domain $E_{⊥}(x,z)=F^{-1}[{\tilde{E}}_{⊥}(k_{x},z)exp(i\cdot k_{z}\Delta z)]$.

**Figure 13:**
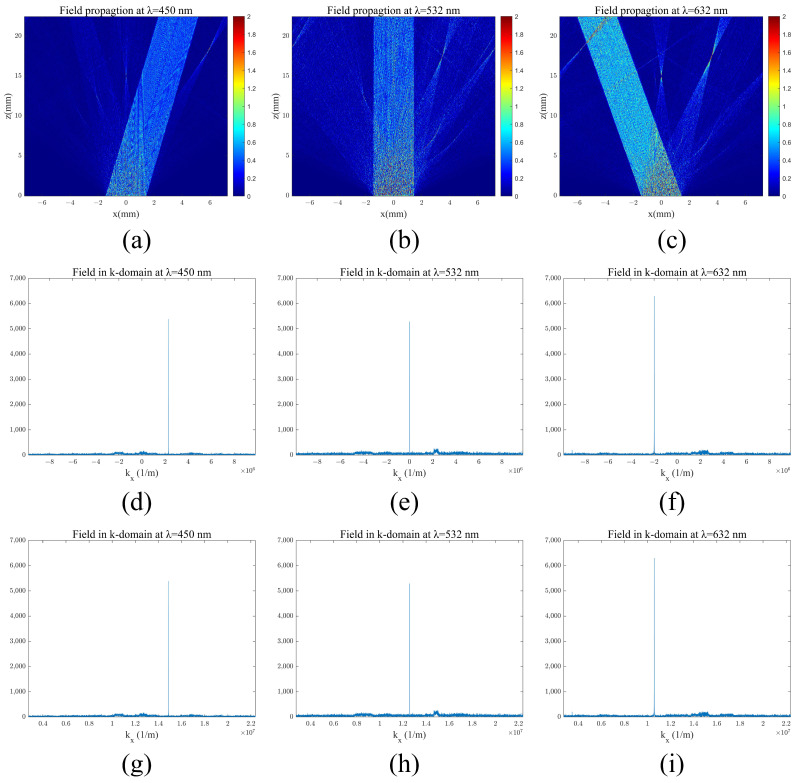
In-coupling light shaping results: (**a**–**c**) field propagation coupled with the metasurface for wavelengths of 450 nm, 532 nm, and 632 nm; (**d**–**f**) amplitude of the field coupled with the metasurface in the k-domain for wavelengths of 450 nm, 532 nm, and 632 nm; (**g**–**i**) amplitude of the field couped with the metasurface and grating in the k-domain for wavelengths of 450 nm, 532 nm, and 632 nm.

Using these three steps, the field results are calculated as several propagation distances, as shown in Figure 13a–c, for the field modulated by the metasurface layer of the in-coupling double-layer DOE.

The component of the wave vector $k_{x}$ in the x-direction of the beam can also be obtained through frequency domain analysis, allowing for a thorough examination of the light transmission process. A Fourier transform can be applied, where the sharpest peak corresponds to the desired light modulation effect, as illustrated in Figure 13d–f,g–i. The components of the wave vector in the x-direction are identified as $2.290\times 10^{6}\left(1/m\right)$, 0, and $-1.989\times 10^{6}\left(1/m\right)$, respectively. Using the following equation:

(9) $$\theta =\mathrm{asin}\left(\frac{k_{x}}{k}\right)$$

the light modulation directions for the three colors were calculated, yielding angles of 9.44°, 0°, and −11.54°, which align with the defined grating incident angle. According to the grating equation, the wave vector modulation for each color resulted in wave vectors of $1.486\times 10^{7}\left(1/m\right)$, $1.257\times 10^{7}\left(1/m\right)$, and $1.058\times 10^{7}\left(1/m\right)$, all corresponding to an angle of 36.236°. This matches the expected propagation angle within the waveguide: $\theta_{\mathrm{wg}}$ = 36.236°. Frequency domain analysis confirmed the modulation capability of the dual-layer coupled incident light information.

**Figure 14:**
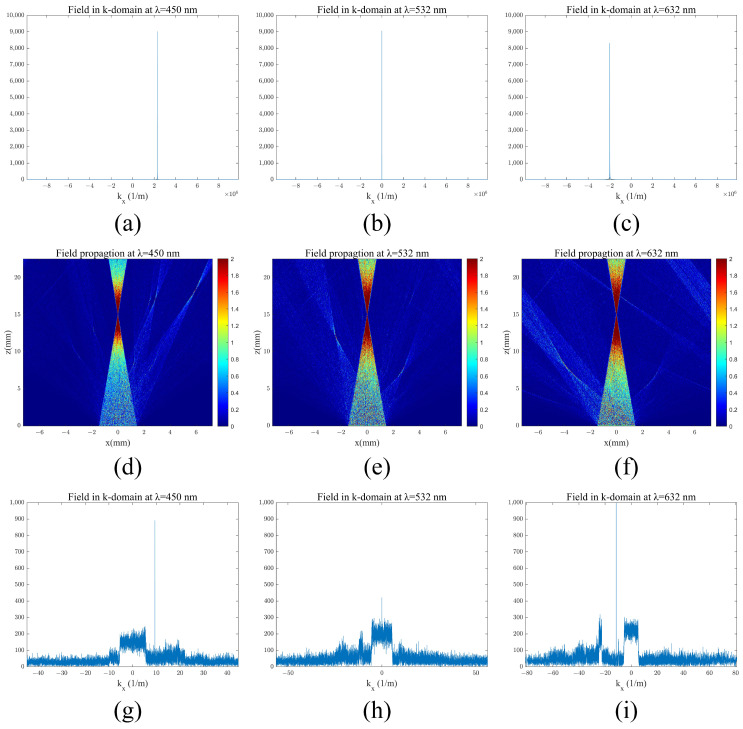
Out-coupling light shaping results: (**a**–**c**) amplitude of field coupled with grating in the k-domain for wavelengths of 450 nm, 532 nm, and 632 nm; (**d**–**f**) field propagation coupled with the metasurface for wavelengths of 450 nm, 532 nm, and 632 nm; (**g**–**i**) amplitude of field couped with grating and metasurface in the k-domain for wavelengths of 450 nm, 532 nm, and 632 nm.

Similarly, for the coupling exit process, when light enters the coupling output grating at $\theta_{\mathrm{wg}}$ = 36.236°, the diffraction angles are $\theta_{\mathrm{out}}\left(\lambda_{R}\right)$ = 9.44°, $\theta_{\mathrm{out}}\left(\lambda_{G}\right)$ = 0°, and $\theta_{\mathrm{out}}\left(\lambda_{B}\right)$ = −11.54°. The field results were calculated as several propagation distances, as shown in Figure 14d–f, for the field modulated by the metasurface layer of the out-coupling double-layer DOEs.

After modulation via the metasurface, an analysis of the optical field, depicted alongside the frequency domain data in Figure 14g–i, revealed that the wavefront phase was restored to the spherical phase. However, certain higher-diffraction orders appeared, suggesting potential room for improvement through the optimization of metasurface unit structures and design parameters. Integrating both coupled incident and exit features in dual-layer diffraction optical elements would enable wavefront modulation collimation and restoration functionality.

### 3.3. Realistic Fabrication Procedure

The pillar, with TiO_2_ as the material, needs to be fabricated using the following steps: 1. use TiO_2_ to coat the substrate SiO_2_; 2. coat TiO_2_ with Cr; 3. coating photoresistent; 4. use electron beam lithography (EBL) exposure to develop the pillar structure; 5. dry-etch Cr using inductively coupled plasma (ICP); 6. clean the photoresistent material on the Cr; 7. dry-etch TiO_2_ using reactive ion beam etching (RIBE); 8. clean the Cr mask using a Ce(NH_4_)_2_(NO_3_) solution, which is reacted with Cr but not reacted with TiO_2_. Using this process, the EBL could be changed into a nanoimprint. Alternatively, the other lithography method could be used.

### 3.4. Simulation of Metasurface-Based Double-Layer Optical Waveguide Imaging Process

**Figure 15:**
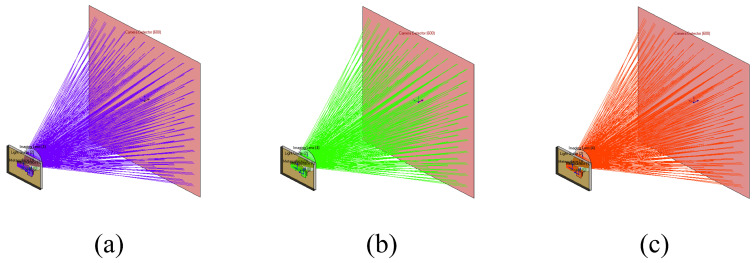
Optical system of metasurface-based double-layer diffractive waveguide combiner for simulation: (**a**) wavelength of 450 nm; (**b**) wavelength of 532 nm; (**c**) wavelength of 632 nm.

**Figure 16:**
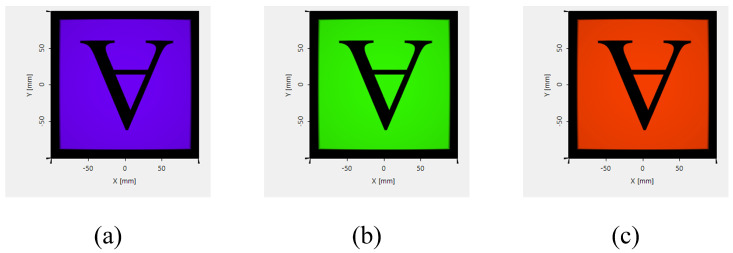
Field-tracing result of optical system: (**a**) wavelength of 450 nm; (**b**) wavelength of 532 nm; (**c**) wavelength of 632 nm.

The optical software conducts a physical optics simulation modeling analysis of a chromatically corrected, bilayer, coupled diffraction waveguide based on a metasurface, as illustrated in Figure 15. The ray-tracing diagrams for the three wavelengths clearly depict the process of optical information transfer. A 200 mm lens is positioned 10 mm after the output coupling of the waveguide. The imaging results at the lens focus, shown in Figure 16, indicate a similar transmission efficiency for the three colors of light. This implies that the chromatically corrected, bilayer, coupled diffraction waveguide based on the metasurface can achieve uniform efficiency in terms of color image transmission. From a simulation perspective, this validates the imaging functionality of the bilayer coupled chromatic correction based on the metasurface. An analysis of the uniformity of the brightness in the imaging results confirms that it meets the defined criteria.

The field of view could be calculated from the focal length of the image lens and the image size on the back focal plane of the image lens. With an image focal length of 200 mm and image size of 177.63 mm × 177.63 mm, the field of view (FOV) is calculated as 47.889° × 47.889°.

The uniformity of the image could represent the transmission efficiency of the diffractive waveguide system at different angular spectra. Using the multi-points method, several points are selected to analyze the uniformity of the image. The nine points are the center of the sub-areas when the image is divided into 3 × 3 sub-areas. The other four points are the corners of the image. The intensity at the 13 points could be used to analyze the uniformity of the image. The simulation results of the energy at different areas of the image is shown in Table 4 for 13 points, along with its relative positions. From these nine test points, the uniformity of the image is determined to be 91.84% and from thirteen test points, the uniformity of the image is determined to be 85.68%.

## 4. Conclusions

This paper presents a double-layer coupled diffractive optical element based on metasurfaces and gratings. It eliminates the dispersion effects associated with single-layer grating coupling. This design achieves the wavefront modulation of optical information and aligns polychromatic light information in a specific direction.

The study validates the imaging capabilities of this optical element in diffractive waveguides, enabling the efficient transmission of polychromatic optical information comparable to single-layer waveguides. The uniformity of the field efficiency for monochromatic light information exceeds 90%, while the transmission efficiency for polychromatic light information reaches over 95%.

## Figures and Tables

**Table 1:** The angles of incident light information and transmission angles within optical waveguides under varying periods.

d	$\theta_{\mathrm{in}}\;(450\;\mathrm{nm})$	$\theta_{\mathrm{in}}\;(532\;\mathrm{nm})$	$\theta_{\mathrm{in}}\;(632\;\mathrm{nm})$	$\theta_{\mathrm{wg}}$
450 nm	10.50°	0°	−12.84°	41.06°
460 nm	10.27°	0°	−12.56°	39.98°
470 nm	10.05°	0°	−12.28°	38.97°
480 nm	9.84°	0°	−12.03°	38.01°
490 nm	9.63°	0°	−11.78°	37.10°
500 nm	9.44°	0°	−11.54°	36.24°

**Table 2:** Structural parameters of in-coupling and out-coupling double-layer DOEs based on metasurface and grating.

DOEs	Metasurface	Grating		
	Pillar Distribution	Slant Angle	Fill Factor	Grating Depth
In-coupling DOE	Figure 9	27°	50%	580 nm
Out-coupling DOE 1	Figure 11a	0°	69%	490 nm
Out-coupling DOE 2	Figure 11b	0°	66%	470 nm
Out-coupling DOE 3	Figure 11c	0°	36%	320 nm

The pillar material is TiO_2_ and substrate material is SiO_2_. Pillar height is 800 nm. Grating material is a lanthanum dense flint glass N-LASF44 (Schott). Grating period was 500 nm.

**Table 3:** Out-coupling efficiencies of exit pupil extension double-layer DOEs.

	Wavelength	450 nm	532 nm	632 nm
1st out-coupling DOE	Grating efficiency	13.10%	13.05%	13.58%
	Metasurface efficiency	59.07%	59.40%	57.07%
	Total efficiency	7.74%	7.75%	7.75%
2nd out-coupling DOE	Grating efficiency	10.27%	12.92%	15.88%
	Metasurface efficiency	71.15%	56.12%	46.15%
	Total efficiency	7.30%	7.25%	7.33%
3rd out-coupling DOE	Grating efficiency	12.32%	12.70%	14.09%
	Metasurface efficiency	61.92%	60.07%	54.02%
	Total efficiency	7.63%	7.65%	7.61%

**Table 4:** Uniformity test data and results of the meta-based double-layer coupled diffractive waveguide.

Relative Position	Intensity	Relative Position	Intensity
(−0.66, 0.66)	$1.561\times 10^{-4}{(V/m)}^{2}$	(0.0, 0.66)	$1.695\times 10^{-4}\;{(V/m)}^{2}$
(−0.66, 0.0)	$1.693\times 10^{-4}\;{(V/m)}^{2}$	(0.0, 0.0)	$1.839\;\times 10^{-4}{(V/m)}^{2}$
(−0.66, −0.66)	$1.563\;\times 10^{-4}\;{(V/m)}^{2}$	(0.0, −0.66)	$1.694\;\times 10^{-4}\;{(V/m)}^{2}$
(0.66, 0.66)	$1.563\;\times 10^{-4}{(V/m)}^{2}$	(0.66, 0.0)	$1.695\;\times 10^{-4}{(V/m)}^{2}$
(0.66, −0.66)	$1.562\;\times 10^{-4}\;{(V/m)}^{2}$		
Uniformity from 9 points		91.84%	
(−0.95, 0.95)	$1.378\;\times 10^{-4}{(V/m)}^{2}$	(0.95, 0.95)	$1.379\;\times 10^{-4}\;{(V/m)}^{2}$
(−0.95, −0.95)	$1.379\;\times 10^{-4}\;{(V/m)}^{2}$	(0.95, −0.95)	$1.378\;\times 10^{-4}\;{(V/m)}^{2}$
Uniformity from 13 points		85.68%	

## Data Availability

The data underlying the results presented in this paper are not publicly available at this time but may be obtained from the authors upon reasonable request. Please email zhangjiahang17@mails.ucas.ac.cn.
